# LDL cholesterol level as a risk factor for retinopathy and nephropathy in children and adults with type 1 diabetes mellitus: A nationwide cohort study

**DOI:** 10.1111/joim.13212

**Published:** 2020-12-27

**Authors:** B. Rathsman, J. Haas, M. Persson, J. Ludvigsson, A.‐M. Svensson, M. Lind, M. Andersson Franko, T. Nyström

**Affiliations:** ^1^ From the Department of Clinical Science and Education Karolinska Institutet Stockholm Sweden; ^2^ Sachs’ Children and Youth Hospital Stockholm Sweden; ^3^ Department of Medicine Clinical Epidemiological Unit Karolinska Institutet Stockholm Sweden; ^4^ Division of Paediatrics Department of Biomedical and Clinical Sciences Linköping University Linköping Sweden; ^5^ Crown Princess Victoria Children’s Hospital Linköping Sweden; ^6^ Department of Molecular and Clinical Medicine Institute of Medicine University of Gothenburg Gothenburg Sweden; ^7^ Centre of Registers in Region Västra Götaland Gothenburg Sweden; ^8^ Department of Medicine NU Hospital Group Uddevalla Sweden

## Abstract

**Background:**

Microvascular complications are common in people with diabetes, where poor glycaemic control is the major contributor. The aim of this study was to explore the association between elevated LDL cholesterol levels and the risk of retinopathy or nephropathy in young individuals with type 1 diabetes.

**Methods:**

This was a nationwide observational population‐based cohort study, including all children and adults with a duration of type 1 diabetes of ≤ 10 years, identified in the Swedish National Diabetes Register between 1998 and 2017. We calculated the crude incidence rates with 95% confidence intervals (CIs) and used multivariable Cox regression to estimate crude and adjusted hazard ratios (HRs) of retinopathy or nephropathy in four LDL cholesterol categories: <2.6 (Reference), 2.6–3.4, 3.4–4.1 and > 4.1 mmol L^−1^.

**Results:**

In total, 11 024/12 350 (retinopathy/nephropathy, both cohorts, respectively) children and adults (median age 21 years, female 42%) were followed up to 28 years from diagnosis until end of study. Median duration of diabetes when entering the study was 6 and 7 years in the retinopathy and nephropathy cohort, respectively. Median LDL cholesterol was 2.4 mmol L^−1^, and median HbA1c level was 61 mmol mol^−1^ (7.7 %). After multivariable adjustment, the HRs (95% CI) for retinopathy in individuals with LDL cholesterol levels of 2.6–3.4, 3.4–4.1 or > 4.1 mmol L^−1^ were as follows: 1.13 (1.03–1.23), 1.16 (1.02–1.32) and 1.18 (0.99–1.41), compared with the reference. The corresponding numbers for nephropathy were as follows: 1.15 (0.96–1.32), 1.30 (1.03–1.65) and 1.41 (1.06–1.89).

**Conclusions:**

Young individuals with type 1 diabetes exposed to high LDL cholesterol levels have an increased risk of retinopathy and nephropathy independent of glycaemia and other identified risk factors for vascular complications.

## Graphical abstract

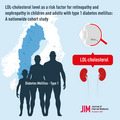

## Introduction

Retinopathy and nephropathy are common microvascular complications in individuals with diabetes. Poor glycaemic control is one major determinant of the timing of a potential microvascular complication, and the progression of microvascular complications is significantly inhibited with improved glycaemic control [1]. Not only risk of microvascular complications is reduced by improved glycaemic control, but also macrovascular complications [2], and there is a strong association between these complications [3]. Poor glycaemic control is, however, not the only predictor of vascular complications [1]. Smoking, obesity, hypertension, and dyslipidaemia are other important risk factors to manage in order to reduce risk of vascular complications in individuals with diabetes [4].

Reducing plasma levels of low‐density lipoprotein (LDL) cholesterol is generally recommended as part of standard care treatment for both primary prevention and secondary prevention of cardiovascular disease [5]. In people with diabetes, there is a paucity of evidence to indicate the age at which lipid‐lowering drugs, that is statins, should be initiated. It was recently suggested in the European Cardiology Society guidelines to delay statin therapy in asymptomatic people with diabetes in the absence of cardiovascular risk factors, or vascular damage until the age of 30 years [6]. The current guidelines by the International Society for Pediatric and Adolescent Diabetes and American Diabetes Association suggest an active approach to dyslipidaemia in childhood, and to screen for dyslipidaemia soon after diagnosis in all children with type 1 diabetes from the age of 11 years [7]. This follows a recommendation of lifestyle modifications in case of LDL cholesterol above 2.6 mmol L^−1^ and that treatment with statins should be considered if such interventions do not lower LDL cholesterol below 3.4 mmol L^−1^ [7].

Large epidemiological studies on the association between dyslipidaemia and risk of microvascular complications in young individuals with type 1 diabetes are scarce. The aim of this nationwide observational cohort study was to explore the association between elevated LDL cholesterol and risk of retinopathy or nephropathy, in young individuals with type 1 diabetes using data from the Swedish national diabetes register.

## Methods

### Study design

This was a nationwide, observational population‐based cohort study. The study was approved by the Swedish Ethical Review Authority (Dnr 977‐17). Participants provided informed consent.

### Study population

The Swedish National Diabetes Registry (NDR) for adults was recently merged with Swediabkids (the paediatric registry for diabetes established in 2000). In the present study, we included information from the NDR for 1998 and onwards, and from Swediabkids for 2000 and onwards, until 31th of December 2017. We included all children, adolescence and adults, with a diagnosis of type 1 diabetes for ten or less years, when first recorded in the registries. The age span in the whole cohort was 0–39 years; that is, patients are included if they are diagnosed with diabetes type 1 before 30 years of age and if duration was <10 years.

Since 1996, the NDR includes information on risk factors, drugs and diabetes complications. In the NDR, type 1 diabetes was defined as treatment with insulin and diagnosis at age 30 years or younger, a definition that has been validated in 97% of cases [8]. In the Swediabkids, more than 97% of Swedish children and adolescents (≤18 years) with type 1 diabetes are registered and consist of outpatient data from all paediatric diabetes centres in Sweden [9]. HLA and autoantibodies are determined in all children and adolescents with newly diagnosed diabetes.

The final data set comprised 26 786 individuals with type 1 diabetes, out of which 15 111 were identified in the Swediabkids, and 19 298 in the NDR. Individuals with at least one registration of retinopathy, and one registration of nephropathy, respectively, and at least one registration on LDL cholesterol and each covariate were included in the final data set. Analyses of risks of retinopathy were based on 11 024 individuals with data on examinations for retinopathy and without missing information on exposure and covariates. The corresponding number for analyses of risks of nephropathy was 12 350 individuals. The overlap between cohorts included 10 262 individuals (Figure S1).

### Endpoints

Endpoints of retinopathy were classified as any retinopathy, preproliferative diabetic retinopathy (PPDR) or proliferative diabetic retinopathy (PDR). Any retinopathy included any signs of retinopathy (simplex retinopathy, PPDR or PDR). PDR was defined as evidence of current proliferations or any earlier laser photocoagulation.

Endpoints of nephropathy were classified as any microalbuminuria defined as two positive test results from three samples taken within one year, with an albumin/creatinine ratio of 3–30 mg mmol^−1^, or urinary albumin of 20–200 μg min^−1^ (20–300 mg L^−1^), or macroalbuminuria defined as an albumin/creatinine ratio >30 mg mmol^−1^ or urinary albumin >200 μg min^−1^ (>300 mg L^−1^).

### Procedures

Individuals were followed from the first observation without missing values in the register (index date) until first event of any retinopathy, or any nephropathy, or until the end of the study 31th of December 2017, whichever came first. On the basis of their index date, patients were assigned to one of the following four LDL cholesterol categories: < 2.6 (Reference), 2.6≤ to <3.4, 3.4≤ to <4.1 and ≥4.1 mmol L^−1^. During follow‐up, LDL cholesterol was assessed using updated mean, enabling study participants to change categories if their LDL cholesterol had improved or worsened; that is, the average of every measurement of covariates, between the current and previous measurement for any of the outcomes, is calculated as a measure of exposure during this interval. If no measurements are made in the interval, the value at the previous examination is used. All other covariates were similarly updated with new measurements that were available during follow‐up. For categorical covariates, the mode was used instead of the mean.

Follow‐up did not end when a mild retinopathy or nephropathy was registered. When all forms of retinopathy or nephropathy were analysed, only the first diagnosis was included; that is, most often mild retinopathy or nephropathy, but when more serious forms were considered milder forms, were ignored and the first diagnosis of the serious form was included.

Analyses of blood lipid levels and HbA1c levels were performed at certified local laboratories. LDL cholesterol values were calculated using the Friedewald formula: LDL cholesterol = total cholesterol – HDL cholesterol – (0.45 × triglycerides), if triglycerides < 4.0 mmol L^−1^ [10]. Analyses of HbA1c are reported according to the International Federation of Clinical Chemistry standard, measured in mmol mol^−1^. We converted all HbA1c values to standard values according to the National Glycohemoglobin Standardization Program [11].

### Co‐linearity of blood lipids

We investigated the possible co‐linearity between different blood lipid profiles. There was a strong co‐linearity observed between LDL cholesterol, total cholesterol (Spearman’s rho 0.85) and non‐HDL‐cholesterol (total cholesterol – HDL cholesterol) levels (Spearman’s rho 0.94), respectively (Figure S2). Hence, non‐HDL cholesterol was not included in the model, primarily because of the high co‐linearity, which made the model unstable, but also since it was superfluous when HDL cholesterol and total cholesterol were included.

### Statistical analysis

Baseline characteristics are based on the first available observation for each individual in the study. Continuous variables are presented as medians and interquartile range (IQR) and categorical variables as proportions. Crude and adjusted hazard ratios (HRs) and confidence intervals (CIs) were estimated using univariable and multivariable Cox regression models with time until endpoints, that is retinopathy or nephropathy, as response variables and time‐varying covariates as exposure variables, that is LDL cholesterol, and potential confounders. The analyses were confined to individuals with data on exposure and covariates and with information on examinations for retinopathy and/or nephropathy. Exposure to risk of development of retinopathy and nephropathy starts when patients are diagnosed with diabetes type 1, which means that duration is the outcome in the Cox regression analyses. Instead, age at diabetes onset was included as a covariate. We also used retinopathy and nephropathy, respectively, as time‐varying covariates when analysing possible competing risks. All covariates that were adjusted for in the final model are shown in Table 1. When no observations of a covariate had been made, the value at the previous examination was used. Only individuals with at least one observation on each covariate were included in the analyses. We did not replace missing values by multiple imputation but chose to exclude patients with missing values.

**Table 1:** All covariates that were adjusted for in the final model

	Retinopathy analyses			Nephropathy analyses		
	Hazard ratio	Confidence interval	*P*‐value	Hazard ratio	Confidence interval	*P*‐value
LDL < 2.6, mmol L^−1^	REF			REF		
2.6 ≤ LDL < 3.4, mmol L^−1^	1.13	1.03–1.23	0.008	1.15	0.96–1.37	0.12
3.4 ≤ LDL < 4.1, mmol L^−1^	1.16	1.02–1.32	0.021	1.30	1.03–1.65	0.030
LDL ≥ 4.1, mmol L^−1^	1.18	0.99–1.41	0.060	1.41	1.05–1.89	0.022
HDL < 1.1, mmol L^−1^	REF			REF		
1.1 ≤ HDL, mmol L^−1^	0.92	0.83–1.01	0.096	0.86	0.72–1.03	0.091
Total cholesterol < 4.5, mmol L^−1^	REF			REF		
Total cholesterol ≥ 4.5, mmol L^−1^	0.89	0.81–0.97	0.008	0.97	0.81–1.16	0.72
Triglyceride < 1.7, mmol L^−1^	REF			REF		
1.7 ≤ Triglyceride, mmol L^−1^	1.18	1.07–1.29	0.001	1.43	1.21–1.68	<0.001
HbA1c < 48, mmol mol^−1^	REF			REF		
48 ≤ HbA1c < 58, mmol mol^−1^	0.94	0.84–1.07	0.35	1.06	0.81–1.37	0.69
58 ≤ HbA1c < 70, mmol mol^−1^	1.15	1.03–1.29	0.016	1.14	0.89–1.46	0.31
70 ≥ HbA1c, mmol mol^−1^	1.46	1.30–1.64	<0.001	1.85	1.45–2.35	<0.001
Male	REF			REF		
Female	1.04	0.98–1.11	0.22	1.33	1.17–1.51	<0.001
Age at diabetes onset, 0–10 years	REF			REF		
Age at diabetes onset, 10–15 years	1.54	1.39–1.71	<0.001	1.46	1.20–1.77	<0.001
Age at diabetes onset, 15–20 years	1.96	1.72–2.23	<0.001	2.12	1.69–2.67	<0.001
Age at diabetes onset, 20–25 years	2.06	1.79–2.36	<0.001	2.00	1.56–2.56	<0.001
Age at diabetes onset, 25–30 years	1.99	1.71–2.32	<0.001	2.04	1.56–2.67	<0.001
BMI^a^ normal 18.5–24.9, kg m^−2^	REF			REF		
BMI^a^ overweight 25–29.9, kg m^−2^	1.10	1.03–1.18	0.005	0.82	0.70–0.95	0.008
BMI^a^ obese ≥ 30, kg m^−2^	1.15	1.04–1.27	0.009	1.01	0.84–1.23	0.89
Nonsmoker	REF			REF		
Smoker	1.32	1.20–1.46	<0.001	1.28	1.06–1.53	0.010
Physical activity, daily	REF			REF		
Physical activity, 3–5 times week^−1^	1.04	0.95–1.13	0.42	0.78	0.65–0.94	0.010
Physical activity, 1–2 times week^−1^	0.99	0.90–1.09	0.89	0.99	0.82–1.19	0.89
Physical activity, <1 times week^−1^	1.08	0.97–1.21	0.14	1.02	0.83–1.26	0.83
Physical activity, never	1.01	0.89–1.15	0.89	1.21	0.96–1.52	0.10
Insulin method, injection	REF			REF		
Insulin method, pump	0.97	0.90–1.04	0.40	0.94	0.81–1.10	0.46
ASA, no	REF			REF		
ASA, yes	1.46	1.10–1.94	0.009	1.35	0.89–2.04	0.15
Antihypertensive, no	REF			REF		
Antihypertensive, yes	1.04	0.91–1.20	0.57	4.45	3.72–5.32	<0.001
Lipid‐lowering drug, no	REF			REF		
Lipid‐lowering drug, yes	1.10	0.96–1.26	0.19	0.96	0.75–1.23	0.73
Normal blood pressure Systolic blood pressure < 140 mmHg	REF			REF		
Hypertension Systolic blood pressure > 140 mmHg	1.15	1.00–1.33	0.057	1.21	0.94–1.55	0.14
90 ≥ eGFR, mL min^−1^	REF			REF		
60 ≤ eGFR < 90, mL min^−1^	0.89	0.82–0.98	0.013	0.92	0.76– 1.11	0.39
45 ≤ eGFR < 60, mL min^−1^	0.87	0.47–1.63	0.67	4.11	2.07–8.16	<0.001
30 ≤ eGFR < 45, mL min^−1^	0.67	0.25–1.82	0.43	5.28	2.41–11.56	<0.001
eGFR < 30, mL min^−1^	0.84	0.21–3.41	0.81	6.20	1.89–20.32	0.003

ASA, acetylsalicylic acid; BMI, body mass index; eGFR, estimated glomerular filtration rate; HbA1c, glycated haemoglobin 1c; HDL cholesterol, high‐density lipoprotein cholesterol; LDL cholesterol, low‐density lipoprotein cholesterol.

^a^ isoBMI was calculated for individuals with age under 18 years according to reference [14]. Continuous variables are presented as medians and interquartile range and categorical variables as proportions.

Since retinopathy and nephropathy most often are symptom‐free, these conditions can only be discovered at an examination. This means that the outcome is interval censored; that is, the exact time point of the outcome can occur anywhere between a negative and a positive examination. It was assumed that a milder complication precedes a serious complication even if it is not registered. Cox regression for interval censored time‐to‐event is not well defined so we used the following algorithm. First, we simulated 1000 data sets where the survival times were uniformly sampled between the time point of the last negative examination and the time point of the first positive examination. Then, Cox regressions were carried out on all data sets and coefficients and standard errors were summarized to account for both estimation error in each model and censoring error between models [12].

Schoenfeld residuals were used to test the assumption of proportional hazard in the Cox regressions. It was not rejected for retinopathy (*P* = 0.089), but for nephropathy (*P* = 0.021), where it was violated with respect to age at onset. A stratified analysis with separate hazard functions in the age at onset strata showed no major change in the HRs with respect to LDL cholesterol.

Survival curves are calculated from the simulated data sets and time‐varying covariates to reflect the assumptions behind the Cox regressions [13]. Stratified analyses with respect to sex, age at diabetes onset, HbA1c, BMI (isoBMI was calculated for individuals with age under 18 years [14]), smoking status and method for insulin administration are presented as estimates and CIs in Forest plots with p‐values for tests of possible interactions. To further investigate the impact of LDL cholesterol on the risk of retinopathy and nephropathy, Cox regressions with 4‐knot spline components were carried out.

## Results

### Patients’ characteristics

Baseline characteristics of the study participants in the retinopathy and nephropathy cohorts, respectively, are shown in Table 2, after stratification into different LDL cholesterol categories in Table S2 and Table S3 (Supplemental Material). The median duration of diabetes before entering the study was 6 and 7 years, respectively. The median age (both cohorts) was 21 years (female 42%), and the median age at onset of type 1 diabetes was 16 and 15 years, in the retinopathy and nephropathy cohorts, respectively (Table 2). In both cohorts, the median LDL cholesterol level was 2.4 mmol L^−1^, and the median HbA1c level was 61 mmol mol^−1^. Proportions of individuals in different LDL cholesterol categories are shown in Table 2. In the retinopathy cohort, 2.8% (312/11023) were treated with lipid‐lowering agents, and corresponding proportion was 2.5% (304/12350) in the nephropathy cohort (Table 2).

**Table 2:** Baseline characteristics of all patients divided in the retinopathy and nephropathy cohort, respectively

	Excluded patients	Retinopathy analysis	Nephropathy analysis
Number	13665	11023	12350
Age, years	12 (7–22)	21 (19–26)	21 (19–25)
Females	44.7%	42.0%	42.8%
Males	55.3%	58.0%	57.2%
Age at diabetes onset, years	11 (6–17)	16 (11–21)	15 (10–21)
Age at diabetes onset, 0–10 years	44.7%	19.8%	23.8%
Age at diabetes onset, 10–15 years	25.3%	26.4%	25.9%
Age at diabetes onset, 15–20 years	11.5%	23.0%	21.5%
Age at diabetes onset, 20–25 years	10.7%	18.4%	17.3%
Age at diabetes onset, 25–30 years	7.8%	12.3%	11.6%
Duration, years	0.2 (0.1–5)	6 (3–9)	7 (3–10)
Follow‐up time, years	3.2 (0.9–6.9)	8.2 (4.8–11.2)	8.5 (4.9–12.0)
Number of LDL cholesterol measurements per individual	0 (0–1)	4 (2–7)	4 (2–7)
LDL cholesterol, mmol L^−1^	2.39 (1.92–2.91)	2.42 (1.98–2.93)	2.42 (1.97–2.95)
LDL cholesterol < 2.6	60.7%	60.3%	59.5%
2.6 ≤ LDL cholesterol < 3.4	27.4%	28.0%	28.2%
3.4 ≤ LDL cholesterol < 4.1	8.3%	8.4%	8.7%
4.1 ≤ LDL cholesterol	3.7%	3.3%	3.6%
HDL cholesterol, mmol L^−1^	1.5 (1.2–1.8)	1.4 (1.2–1.7)	1.4 (1.2–1.7)
HDL cholesterol < 1.1	11.8%	13.3%	13.2%
1.1 ≤ HDL cholesterol	88.2%	86.7%	86.8%
Total cholesterol, mmol L^−1^	4.4 (3.8–4.9)	4.4 (3.8–5.0)	4.4 (3.8–5.0)
Total cholesterol < 4.5	54.8%	55.1%	54.4%
Total cholesterol ≥ 4.5	45.2%	44.9%	45.6%
Triglyceride, mmol L^−1^	0.90 (0.60–1.30)	0.90 (0.62–1.26)	0.90 (0.65–1.29)
Triglyceride < 1.7	85.7%	86.5%	86.2%
1.7 ≤ Triglyceride	14.3%	13.5%	13.8%
HbA1c, mmol mol^−1^	58 (49–70)	61 (51–71)	61 (52–72)
HbA1c < 48	21.2%	17.0%	16.6%
48 ≤ HbA1c < 58	27.6%	24.7%	23.8%
58 ≤ HbA1c < 70	25.8%	30.0%	30.6%
70 ≤ HbA1c	25.4%	28.3%	29.1%
BMI^a^, kg m^−2^	18.9 (16.6–23.1)	23.4 (21.4–26.1)	23.2 (21.1–26.0)
Normal 18.5–24.9	83.1%	65.4%	66.3%
Overweight 25–29.9	11.3%	25.6%	25.0%
Obese ≥ 30	5.6%	9.0%	8.7%
Nonsmokers	95.2%	91.4%	91.5%
Smokers	4.8%	8.6%	8.5%
Physical activity; daily	22.2%	17.7%	17.3%
Physical activity; 3–5 times week^−1^	31.1%	32.9%	33.7%
Physical activity; 1–2 times week^−1^	31.7%	26.8%	27.3%
Physical activity; <1 times week^−1^	8.3%	14.0%	13.4%
Physical activity; never	6.6%	8.6%	8.3%
Insulin method; injection	81.4%	81.2%	79.3%
Insulin method; pump	18.6%	18.8%	20.7%
ASA; no	98.9%	99.2%	99.3%
ASA; yes	1.1%	0.8%	0.7%
Antihypertensive; no	95.6%	96.7%	97.2%
Antihypertensive; yes	4.4%	3.3%	2.8%
Lipid‐lowering drug; no	97.5%	97.2%	97.5%
Lipid‐lowering drug; yes	2.5%	2.8%	2.5%
Normal blood pressure, Systolic blood pressure < 140 mmHg	95.8%	96.4%	95.9%
Hypertension, Systolic blood pressure > 140 mmHg	4.2%	3.6%	4.1%
eGFR, mL min^−1^	109 (92–131)	111 (97–128)	111 (98–128)
eGFR < 30	0.14%	0.07%	0.03%
30 ≤ eGFR < 45	0.17%	0.05%	0.06%
45 ≤ eGFR < 60	0.83%	0.25%	0.23%
60 ≤ eGFR < 90	21.44%	14.15%	13.63%
eGFR ≥ 90	77.42%	85.47%	86.06%

ASA, acetylsalicylic acid; BMI, body mass index; eGFR, estimated glomerular filtration rate; HbA1c, glycated haemoglobin 1c; HDL cholesterol, high‐density lipoprotein cholesterol; LDL, cholesterol, low‐density lipoprotein cholesterol.

Yrs, years.

^a^ isoBMI was calculated for individuals with age under 18 years according to reference [14]. Continuous variables are presented as medians and interquartile range and categorical variables as proportions.

### Retinopathy in relation to LDL cholesterol levels

**Fig. 1:**
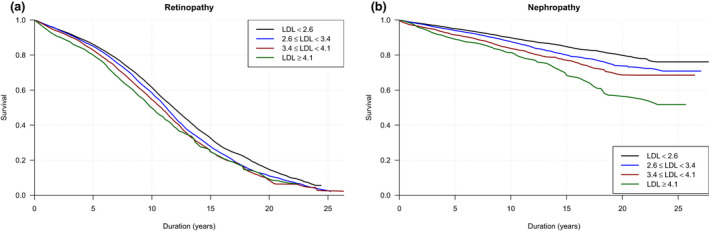
Crude survival curves illustrated the accumulated risk of retinopathy (a) and nephropathy (b) based on these observed time intervals in young people with type 1 diabetes. (low‐density lipoprotein, LDL)

The estimated retinopathy survival curves are shown in Figure 1A. The median follow‐up time was 8.2 (IQR 4.8–11.2) years. The event rate of any retinopathy in each LDL cholesterol categories at <2.6, 2.6–3.4, 3.4–4.1 and ≥4.1 mmol L^−1^ was as follows: 6.77 (IQR 6.49–7.05), 7.76 (IQR 7.34–8.18), 8.61 (IQR 7.80–9.43) and 9.21 (IQR 7.82–10.60), per 100 person‐years, respectively (Table 3).

**Table 3:** Number of events, event rate and relative risks for any retinopathy, that is mild to severe retinopathy, and nephropathy, that is microalbuminuria or macroalbuminuria, in children and adults with type 1 diabetes

Exposure (mmol L^−1^)	Events (*n*)	Event rate 100 person‐yrs	Crude HR	HR adjusted for sex and age	HR adjusted
Retinopathy^a^					
LDL < 2.6	2259	6.77 (6.49–7.05)	REF	REF	REF
2.6 ≤ LDL < 3.4	1295	7.76 (7.34–8.18)	1.15 (1.07–1.23) *P* < 0.001	1.12 (1.04–1.20) *P* = 0.002	1.13 (1.03–1.23) *P* = 0.008
3.4 ≤ LDL < 4.1	429	8.61 (7.80–9.43)	1.26 (1.14–1.40) *P* < 0.001	1.21 (1.09–1.34) *P* < 0.001	1.16 (1.02–1.32) *P* = 0.021
LDL ≥ 4.1	168	9.21 (7.82–10.60)	1.37 (1.17–1.60) *P* < 0.001	1.33 (1.13–1.55) *P* < 0.001	1.18 (0.99–1.41) *P* = 0.060
PPDR/PDR/laser photocoagulation vs. non/simplex					
LDL < 2.6	171	0.38 (0.32–0.43)	REF	REF	REF
2.6 ≤ LDL < 3.4	124	0.53 (0.44–0.62)	1.39 (1.11–1.76) *P* = 0.005	1.34 (1.06–1.69) *P* = 0.014	1.02 (0.76–1.37) *P* = 0.90
3.4 ≤ LDL < 4.1	41	0.56 (0.39–0.73)	1.42 (1.01–2.01) *P* = 0.046	1.29 (0.92–1.82) *P* = 0.14	0.82 (0.55–1.24) *P* = 0.34
LDL ≥ 4.1	18	0.63 (0.34–0.92)	1.61 (0.99–2.61) *P* = 0.054	1.53 (0.94–2.49) *P* = 0.087	0.80 (0.47–1.38) *P* = 0.42
PDR/laser photocoagulation vs. non/simplex/PPDR					
LDL < 2.6	53	0.12 (0.08–0.15)	REF	REF	REF
2.6 ≤ LDL < 3.4	33	0.14 (0.09–0.19)	1.20 (0.77–1.85) *P* = 0.41	1.11 (0.72–1.72) *P* = 0.64	0.90 (0.52–1.56) *P* = 0.71
3.4 ≤ LDL < 4.1	13	0.18 (0.08–0.27)	1.46 (0.79–2.68) *P* = 0.22	1.28 (0.70–2.36) *P* = 0.43	0.94 (0.45–1.96) *P* = 0.87
LDL ≥ 4.1	7	0.25 (0.06–0.43)	2.00 (0.91–4.41) *P* = 0.085	1.83 (0.83–4.04) *P* = 0.13	1.10 (0.44–2.75) *P* = 0.84
Nephropathy^b^					
LDL < 2.6	513	1.11 (1.02–1.21)	REF	REF	REF
2.6 ≤ LDL < 3.4	334	1.44 (1.29–1.60)	1.30 (1.13–1.49) *P* < 0.001	1.25 (1.09–1.43) *P* = 0.001	1.15 (0.96–1.37) *P* = 0.12
3.4 ≤ LDL < 4.1	132	1.79 (1.49–2.10)	1.61 (1.33–1.94) *P* < 0.001	1.53 (1.27–1.86) *P* < 0.001	1.30 (1.03–1.65) *P* = 0.030
LDL ≥ 4.1	69	2.45 (1.87–3.03)	2.20 (1.71–2.82) *P* < 0.001	2.07 (1.61–2.66) *P* < 0.001	1.41 (1.05–1.89) *P* = 0.022
Microalbuminuria					
LDL < 2.6	483	1.04 (0.94–1.13)	REF	REF	REF
2.6 ≤ LDL < 3.4	310	1.33 (1.18–1.48)	1.28 (1.11–1.48) *P* = 0.001	1.24 (1.07–1.43) *P* = 0.004	1.16 (0.97–1.39) *P* = 0.11
3.4 ≤ LDL < 4.1	124	1.64 (1.35–1.93)	1.57 (1.29–1.92) *P* < 0.001	1.51 (1.24–1.84) *P* < 0.001	1.30 (1.02–1.66) *P* = 0.035
LDL ≥ 4.1	64	2.30 (1.74–2.86)	2.20 (1.70–2.86) *P* < 0.001	2.07 (1.60–2.69) *P* < 0.001	1.46 (1.08–1.98) *P* = 0.015
Macroalbuminuria					
LDL < 2.6	85	0.18 (0.14–0.22)	REF	REF	REF
2.6 ≤ LDL < 3.4	64	0.26 (0.20–0.33)	1.47 (1.06–2.03) *P* = 0.020	1.41 (1.02–1.95) *P* = 0.038	1.04 (0.69–1.58) *P* = 0.85
3.4 ≤ LDL < 4.1	27	0.35 (0.22–0.48)	1.95 (1.26–3.01) *P* = 0.003	1.83 (1.18–2.82) *P* = 0.007	1.25 (0.73–2.13) *P* = 0.42
LDL ≥ 4.1	18	0.59 (0.32 −0.86)	3.29 (1.98–5.47) *P* < 0.001	3.09 (1.85–5.14) *P* < 0.001	1.79 (0.97–3.28) *P* = 0.062

HR; hazard ratio; PDR; proliferative diabetes retinopathy; PPDR; preproliferative diabetes retinopathy.

^a^ Defined as any of; simplex retinopathy/PPDR/PDR/laser.

^b^ Defined as any of microalbuminuria or macroalbuminuria.

In the unadjusted analysis, there was a significant association between increased LDL cholesterol levels and risk of retinopathy. After adjustment for sex and age, individuals with LDL cholesterol above 2.6 mmol L^−1^ had a significantly higher risk of retinopathy compared with individuals with LDL cholesterol below 2.6 mmol L^−1^ (Table 3). After multivariable adjustments, the HR (95% CI) for any retinopathy was as follows: 1.13 (1.03–1.23), 1.16 (1.02–1.32) and 1.18 (0.99–1.41) in subjects exposed to 2.6–3.4, 3.4–4.1 and above 4.1 mmol L^−1^, respectively, compared with individuals exposed to LDL cholesterol below 2.6 mmol L^−1^ (Table 3). There was no significant interaction between LDL cholesterol and sex, age at diabetes onset, HbA1c, BMI, smoking or method used for insulin administration, for the risk of retinopathy (Figure S2A, Supplemental Material). The presence of nephropathy was not significant (*P* = 0.98) when included as a covariate, indicating no competing risk between retinopathy and nephropathy.

### Comparison of different degrees of retinopathy in relation to LDL cholesterol levels

We further assigned individuals to different degrees of retinopathy to compare severe retinopathy with milder degrees: PPDR/PRD/laser vs. simplex retinopathy and PRD/laser vs. simplex retinopathy/PPDR, respectively, into the same LDL cholesterol categories (Table 3).

Number of events and event rate was much less for severe retinopathy compared with mild retinopathy. The proportion for PPDR/PRD/laser vs. simplex retinopathy was 6.8% (416/6079), and for PRD/laser vs. simplex retinopathy/PDR 2.5% (155/6340), respectively (Table 3). In the categories 2.6–3.4 and 3.4–4.1 mmol L^−1^, there was an increased risk for PPDR/PRD/laser, HR (95% CI) 1.39 (1.11–1.76) and HR (95% CI) 1.42 (1.02–2.01), respectively, in the unadjusted analysis, and after adjustment for sex and age in the category 2.6–3.4 mmol L^−1^, HR (95% CI) 1.34 (1.06–1.69), compared with simplex or no retinopathy. After multivariable adjustments, there was no significant difference between risks of mild and severe retinopathies in the different categories of LDL cholesterol (Table 3).

### Nephropathy in relation to LDL cholesterol levels

The estimated nephropathy survival curves are shown in Figure 1B. The median follow‐up time was 8.5 (IQR 4.9–12.0) years. The event rate of nephropathy in individuals with index LDL cholesterol <2.6, 2.6–3.4, 3.4–4.1 and >4.1 mmol L^−1^ was as follows: 1.11 (CI 1.02–1.21), 1.44 (CI 1.29–1.60), 1.79 (CI 1.49–2.10) and 2.45 (CI 1.87–3.03), per 100 person‐years, respectively (Table 3).

In the unadjusted analysis, there was a significant association between increased LDL cholesterol and nephropathy (Table 3). After adjustment for sex and age, individuals with high LDL cholesterol levels had a significantly higher risk of nephropathy compared with individuals with LDL cholesterol < 2.6 mmol L^−1^. After multivariable adjustments, the HRs (95% CI) for nephropathy were as follows: 1.15 (0.96–1.37), 1.30 (1.03–1.65) and 1.41 (1.05–1.89) in individuals exposed to: 2.6–3.4, 3.4–4.1 and ≥4.1 mmol L^−1^, respectively, compared with individuals exposed to LDL cholesterol below 2.6 mmol L^−1^ (Table 3). There was no significant interaction between LDL cholesterol and sex, age at diabetes onset, HbA1c, BMI, smoking or insulin administration method used, for the risk of nephropathy (Figure S2B). The presence of retinopathy was not significant (*P* = 0.35) when included as a covariate, further indicating no competing risk between retinopathy and nephropathy.

For the different degrees of nephropathy, that is microalbuminuria and macroalbuminuria, the number of events and event rate for macroalbuminuria was much lower compared with events of microalbuminuria (Table 3). After multivariable adjustments, the HRs (95% CI) for microalbuminuria were as follows: 1.16 (0.97–1.39), 1.30 (1.02–1.66) and 1.46 (1.08–1.98); corresponding numbers for macroalbuminuria were HRs (95% CI): 1.04 (0.69–1.58), 1.25 (0.73–2.13) and 1.79 (0.97–3.28) in individuals exposed to 2.6–3.4, 3.4–4.1 and ≥ 4.1 mmol L^−1^, compared with individuals exposed to LDL cholesterol below 2.6 mmol L^−1^ (Table 3).

### LDL cholesterol and risk of retinopathy or nephropathy in individuals free from retinopathy and nephropathy at the first examination

Since the outcomes were interval censored, a sensitive analysis was performed. We excluded all individuals with retinopathy 1162/11023 (10.5%) and nephropathy 415/12350 (3.4%) at the first examination. After multivariable adjustments, the HRs (95% CI) for any retinopathy were as follows: 1.16 (1.05–1.28), 1.17 (1.01–1.36) and 1.12 (0.99–1.39) in subjects exposed to 2.6–3.4, 3.4–4.1 and ≥4.1 mmol L^−1^, respectively, compared with individuals exposed to LDL cholesterol below 2.6 mmol L^−1^. The corresponding numbers for nephropathy were HRs (95% CI): 1.22 (0.97–1.53), 1.31 (0.96–1.77) and 1.30 (0.88–1.92).

### The nonlinear relationship between LDL cholesterol levels and microvascular complications

We also analysed the potential nonlinear relationship between LDL cholesterol levels and risk of retinopathy and nephropathy, by entering LDL cholesterol levels modelled with restricted cubic splines in a multivariable Cox regression model (Figure S3A and B). The HRs (95% CI) for an absolute increase in LDL cholesterol of 0.5 mmol L^−1^ (above 2.6 mmol L^−1^) for retinopathy were HR (95% CI) 1.03 (1.00–1.06), and for nephropathy HR (95% CI) 1.09 (1.04–1.15).

## Discussion

The main finding, in this nationwide cohort follow‐up study of young individuals with type 1 diabetes, is that exposure to LDL cholesterol above the recommended level of 2.6 mmol L^−1^ is significantly associated with an increased risk of retinopathy, and above the level of 3.4 mmol L^−1^ with an increased risk of nephropathy. The association between increased LDL cholesterol levels and risk of microvascular complications remained significantly increased also after consideration of a number of clinical characteristics and other identified risk factors, including glycaemic control.

During the median follow‐up of eight years, a substantial part of the individuals developed retinopathy, and a lesser part of the individuals developed nephropathy. The estimated survival curves of these complications are much in line with a recent longitudinal observational study on people with type 1 diabetes younger than 35 years who were followed for up to 24 years, in which a majority of the individuals developed retinopathy, and a lesser proportion of the individuals developed nephropathy [15]. Not surprisingly, glycaemic control was the main driver for the risk of developing such complications [15], which has been confirmed by multiple studies [1, 16, 17, 18, 19, 20]. Nevertheless, other risk factors than hyperglycaemia are also contributing factors to microvascular complications in type 1 diabetes [4].

Young people with type 1 diabetes face a much higher risk for cardiovascular disease than the general population [21, 22, 23]. It was recently demonstrated that age at onset of type 1 diabetes is an important determinant of survival and risk of cardiovascular disease, which may support early use of cardioprotective drugs in this group [24]. Estimates based on results from population studies indicate that 35% of children with type 1 diabetes have two or more modifiable cardiovascular risk factors [25]. Beside poor glycaemic control, dyslipidaemia is one of the most frequently observed cardiovascular risk factors in this group [25]. In the present study, 40% of the individuals at baseline had LDL cholesterol above the level of 2.6 mmol L^−1^ a proportion similar to previous observations [25, 26]. Studies in young individuals with type 1 diabetes demonstrate increased blood lipids [27], which can be due to insufficient insulin treatment since poor glycaemic control is associated with dyslipidaemia [27, 28]. This may simply explain the association between LDL cholesterol levels and the risk of retinopathy and nephropathy observed in the present study. However, after adjustment for multiple cardiovascular risk factors, including glycaemic control and diabetes duration, there was still an increased risk of retinopathy observed if LDL cholesterol levels exceeded 2.6 mmol L^−1^, and above 3.4 mmol L^−1^ for nephropathy. Interestingly, there was a 16% increased risk of retinopathy observed already in individuals with LDL cholesterol between 2.6 and 3.4 mmol L^−1^, findings that may suggest an even earlier recommendation of statin treatment in young individuals with type 1 diabetes [7]. Other factors often associated with both dyslipidaemia and risk of vascular complications include obesity, hypertension, sedentary lifestyle and smoking [4]. It has also been demonstrated that girls with type 1 diabetes are more likely to have elevated LDL cholesterol levels than boys [26]. Our analyses revealed no significant interactions between these additional risk factors, and all other covariates, and LDL cholesterol for the risk of retinopathy and nephropathy.

Large epidemiological studies on the association between dyslipidaemia and risk of retinopathy in young individuals with type 1 diabetes are scarce, and previous results are inconclusive [16, 18, 19, 29]. In a recent meta‐analysis, slightly increased LDL cholesterol levels were observed in people with type 2 diabetes and newly diagnosed retinopathy [30]. In the Diabetes Control and Complications Trial, it was demonstrated that severity of retinopathy increased inversely with HDL cholesterol levels [18]. The importance of lipid control for the risk of retinopathy and nephropathy was also shown in the Pittsburgh Epidemiology cohort study [31]. Results from these studies are in line with the findings from our large nationwide study.

A number of recent studies have investigated the role of dyslipidaemia for risk of transformation of mild microvascular complications to more advanced stages. The present study was not designed to study the trajectory of microvascular complications. However, we compared mild vascular complications with severe complications in relation to levels of LDL cholesterol exposure over time. After fully adjustments, there was no increased risk of severe retinopathy or severe nephropathy due to the exposure of LDL cholesterol above 2.6 mmol L^−1^, starting out with milder forms of retinopathy and nephropathy. The lower number of severe events compared with milder events may partly explain the similar risks of severe events observed in individuals in the highest LDL cholesterol categories. However, our results support that other factors, than dyslipidaemia, may contribute to the progression of the microvascular complications [4, 32].

In the FinnDiane study, it was recently demonstrated that dyslipidaemia only increased risk of nephropathy in the presence of retinopathy. This in turn suggests a shared pathogenetic background, which could be addressed in terms of prevention [33]. In the current study, there was no significant interaction between increased LDL cholesterol with risk of retinopathy or nephropathy, when using these endpoints as time‐varying covariates for the respective single outcome of interest; this contrasts the finding from the FinnDiane study [33]. Nevertheless, regardless of shared pathogenic mechanism, it is of great importance to treat dyslipidaemia to prevent cardiovascular complications in people at high risk [4]. Recently, in the Adolescent Type 1 Diabetes Cardio‐Renal Intervention Trial (AdDIT) it was investigated whether treatment with angiotensin‐converting enzyme inhibitors and statins may protect against nephropathy [34]. The use of these agents for a period of 2–4 years did not show any effects on the primary endpoint, that is normalizing the albumin‐to‐creatinine ratio over time [34], chosen as a proxy for risk of cardiovascular disease and preterm death [23]. Even though the relative risk of cardiovascular disease is much higher in young people with type 1 diabetes, compared with their matched peers from the general population, the absolute risk of cardiovascular disease is still very low, requiring a large number of individuals to be studied [24]. There is a need for randomized trials to investigate the efficacy and safety of lipid‐lowering drugs in children and young people with type 1 diabetes [4]. Paradoxically, due to the knowledge of the cardiovascular benefits of lowering LDL cholesterol in people with type 2 diabetes, it may even be unethical to randomize people with type 1 diabetes to placebo in such a study.

The primary strength of this study is the large population‐based cohort, followed from childhood to young adulthood, covering close to 100% of individuals with type 1 diabetes in Sweden. Our study has limitations. Since our study excluded all individuals with no registration of examination for retinopathy or nephropathy, respectively, and without any LDL cholesterol measurements, we cannot rule out that these individuals had a worse prognosis. However, the distribution of covariates which may confound our findings was not much different comparing the excluded individuals with the two final cohorts. We lacked information on insulin doses and socio‐economical background. Different lipid profiles may be differently associated with the outcomes of the present study. However, prior to our final analysis we investigated the association between different lipid profiles, which demonstrated high co‐linearity for LDL cholesterol levels and total cholesterol and non‐HDL‐cholesterol levels, respectively. Also, in the final analysis model, after adjustments for levels of HDL cholesterol and triglycerides, the association between LDL cholesterol and the outcomes remained significant, suggesting LDL cholesterol as an independent risk factor for microvascular complications. Since retinopathy and nephropathy were interval censored, the exact time point of the outcome could not be defined. However, the survival times were uniformly sampled in the final statistical model, and after excluding individuals with retinopathy or nephropathy, at their first examination, the proportion of the risk estimates (with reservation for the group in the highest LDL cholesterol category) were much similar to the estimates from the main analysis, which may simply be explained by the low numbers of events in that group. As in any observational study, we cannot draw any conclusion on causality behind exposure and outcomes.

In conclusion, our study demonstrates that young individuals with type 1 diabetes exposed to high LDL cholesterol levels have an increased risk of retinopathy and nephropathy independent of glucose control and other known risk factors for microvascular complications.

## Author contributions

All authors designed the study. MAF performed the statistical analysis. TN, BR, MP, MAF and JH analysed the data. TN, JH and BR drafted the manuscript. All authors reviewed, commented and approved the final version.

## Conflict of interest statement

TN has received honoraria on expert group participation from AstraZeneca, Merck Sharp & Dohme, Novo Nordisk, Eli Lilly and Company, Boehringer Ingelheim, Abbot and Amgen. ML has received grants from Astra Zeneca, Dexcom, Novo Nordisk, been a consultant or received honoraria from Dexcom, Eli Lilly, Medtronic, Novo Nordisk and Rubin Medical and participated in advisory boards for MSD and Novo Nordisk. All other authors had nothing to disclose.

## Funding information

TN is funded by the Swedish Heart‐Lung Foundation (20190298) and has an ALF agreement between Stockholm County Council and Karolinska Institute (20170120). MP is funded by Stockholm City Council, Barndiabetesfonden and Diabetesfonden.

## Author contribution

**Björn Rathsman:** Conceptualization (equal); Data curation (equal); Investigation (equal); Methodology (equal); Writing‐original draft (equal); Writing‐review & editing (equal). **Josephine Haas:** Data curation (supporting); Formal analysis (supporting); Writing‐original draft (supporting); Writing‐review & editing (supporting). **Martina Persson:** Conceptualization (supporting); Data curation (supporting); Formal analysis (supporting); Writing‐review & editing (supporting). **Johnny Ludvigsson:** Conceptualization (supporting); Formal analysis (supporting); Writing‐review & editing (equal). **Ann‐Marie Svensson:** Data curation (equal); Resources (equal); Writing‐review & editing (equal). **Marcus Lind:** Conceptualization (equal); Data curation (equal); Investigation (equal); Writing‐review & editing (equal). **Mikael Andersson Franko:** Conceptualization (equal); Data curation (lead); Investigation (equal); Software (lead); Writing‐review & editing (equal). **Thomas Nyström:** Conceptualization (lead); Data curation (lead); Formal analysis (lead); Funding acquisition (lead); Investigation (lead); Methodology (equal); Project administration (supporting); Resources (equal); Supervision (lead); Validation (lead); Writing‐original draft (lead); Writing‐review & editing (lead).

## Supporting information


**Table S1.** Baseline characteristics of the retinopathy cohort (n=11023) into 4 categories of LDL‐cholesterol levels.
**Table S2.** Baseline characteristics of the nephropathy cohort (n=12350) into 4 categories of LDL‐cholesterol levels.
**Figure S1.** Flowchart for the studied group. The overlap between cohorts included 10,262 individuals.
**Figure S2.** Scatter plots of blood‐lipids on logarithmic scales. Each square contains pairwise measurements made at the same time of two lipids on each patient illustrated.
**Figure S3.** Adjusted hazard ratios for Retinopathy (A) and Nephropathy (B) in young people with type 1 diabetes, according to baseline LDL‐cholesterol level.
**Figure S4.** Relationship between LDL‐cholesterol levels, as a continuous variable, and risk of Retinopathy (A) and Nephropathy (B).
