# Supplementary material for: LDL cholesterol level as a risk factor for retinopathy and nephropathy in children and adults with type 1 diabetes mellitus: A nationwide cohort study
Source: J Intern Med. 2020 Dec 27;289(6):873–86. doi: 10.1111/joim.13212 (PMC8247303; doi:10.1111/joim.13212)
Supplement: Supplementary file 1 — Table S1. Baseline characteristics of the retinopathy cohort (n=11023) into 4 categories of LDL‐cholesterol levels. Table S2. Baseline characteristics of the nephropathy cohort (n=12350) into 4 categories of LDL‐cholesterol levels. Figure S1. Flowchart for the studied group. The overlap between cohorts included 10,262 individuals. Figure S2. Scatter plots of blood‐lipids on logarithmic scales. Each square contains pairwise measurements made at the same time of two lipids on each patient illustrated. Figure S3. Adjusted hazard ratios for Retinopathy (A) and Nephropathy (B) in young people with type 1 diabetes, according to baseline LDL‐cholesterol level. Figure S4. Relationship between LDL‐cholesterol levels, as a continuous variable, and risk of Retinopathy (A) and Nephropathy (B). [file JOIM-289-873-s001.docx]

# **SUPPLEMENTAL MATERIAL**

# **LDL-cholesterol level as a risk factor for retinopathy and nephropathy in children and adults with type 1 diabetes mellitus: A nationwide cohort study**

**Björn Rathsman^1,2^, Josephine Haas^1,2^, Martina Persson^1,2,3^, Johnny Ludvigsson^4,5^, Ann-Marie Svensson^6,7^, Marcus Lind^6,8^, Mikael Andersson Franko^1^, Thomas Nyström^1^**

^1^Department of Clinical Science and Education, Karolinska Institutet, Södersjukhuset, Stockholm, Sweden

^2^Sachs’ Children and Youth Hospital, Södersjukhuset, Stockholm, Sweden

^3^Department of Medicine, Clinical Epidemiological Unit, Karolinska Institutet, Stockholm, Sweden

^4^Division of Paediatrics, Department of Biomedical and Clinical Sciences, Linköping University, Linköping, Sweden

^5^Crown Princess Victoria Children’s Hospital, Region Östergötland, Linköping, Sweden

^6^Department of Molecular and Clinical Medicine, Institute of Medicine, University of Gothenburg, Gothenburg, Sweden

^7^Centre of Registers in Region Västra Götaland, Sweden

^8^Department of Medicine, NU Hospital Group, Uddevalla, Sweden

# **Table S1.** Baseline characteristics of the retinopathy cohort (n=11023) into 4 categories of LDL-cholesterol levels.

|  | LDL<2.6 | 2.6 ≤ LDL< 3.4 | 3.4 ≤ LDL< 4.1 | LDL≥ 4.1 |
| --- | --- | --- | --- | --- |
| Number | 6642 | 3084 | 931 | 366 |
| Age, yrs | 21 (19-25) | 22 (19-27) | 22 (19-28) | 22 (19-28) |
| Females | 40.4% | 44.4% | 43.8% | 47.0% |
| Males | 59.6% | 55.6% | 56.2% | 53.0% |
| Debut age, yrs | 15 (11-21) | 17 (12-23) | 17 (11-23) | 17 (11-24) |
| 0-10 yrs | 21.0% | 17.6% | 19.0% | 18.9% |
| 10-15 yrs | 27.8% | 24.3% | 24.6% | 22.7% |
| 15-20 yrs | 24.0% | 22.7% | 18.5% | 19.9% |
| 20-25 yrs | 17.0% | 21.2% | 19.4% | 18.6% |
| 25-30 yrs | 10.1% | 14.2% | 18.5% | 19.9% |
| Duration, yrs | 6 (3 – 9) | 6 (3-9) | 7 (3-9) | 7 (3 – 9) |
| Follow-up time, yrs | 8.2 (4.8-11.2) | 8.0 (4.7-10.9) | 8.1 (4.7-11.5) | 7.7 (4.2-11.5) |
| Number of LDL-cholesterol measurements per individual | 5 (3 – 7) | 5 (3 – 8) | 5 (3 – 8) | 5 (3 – 8) |
| HDL-cholesterol, mmol/L | 1.5 (1.2 – 1.7) | 1.4 (1.2 – 1.6) | 1.3 (1.1 – 1.6) | 1.3 (1.1 – 1.5) |
| < 1.1 | 11.3% | 14.9% | 19.0% | 21.0% |
| ≥ 1.1 | 88.7% | 85.1% | 81.0% | 79.0% |
| Total-cholesterol, mmol/L | 4.0 (3.6 – 4.3) | 4.8 (4.5 – 5.2) | 5.6 (5.3 – 6.0) | 6.6 (6.2 – 7.1) |
| < 4.5 | 82.3% | 19.5% | 0.5% | 0.3% |
| ≥ 4.5 | 17.7% | 80.5% | 99.5% | 99.7% |
| Triglyceride, mmol/L | 0.8 (0.6 – 1.1) | 1.0 (0.7 – 1.3) | 1.2 (0.9 – 1.7) | 1.4 (1.0 – 2.3) |
| < 1.7 | 90.9% | 84.4% | 72.8% | 59.0% |
| ≥ 1.7 | 9.1% | 15.6% | 27.2% | 41.0% |
| HbA1c, mmol/mol | 59 (50 – 69) | 62 (52 – 73) | 65 (55 – 76) | 69 (56 – 84) |
| < 48 | 18.9% | 15.1% | 11.8% | 12.6% |
| 48 ≤ to < 58 | 26.5% | 22.9% | 20.6% | 16.9% |
| 58 ≤ to < 70 | 30.2% | 30.5% | 29.8% | 21.9% |
| ≥ 70 | 24.4% | 31.5% | 37.8% | 48.6% |
| ^*^BMI (Kg/m^2^) | 23.0 (21.0 – 25.3) | 24.0 (21.8 – 26.8) | 25.0 (22.6 – 28.7) | 25.5 (22.6 – 28.8) |
| Normal 18.5-24.9 | 71.6% | 59.4% | 49.1% | 45.4% |
| Overweight 25-29.9 | 22.6% | 28.9% | 32.4% | 34.2% |
| Obese ≥ 30 | 5.8% | 11.7% | 18.5% | 20.4% |
| Non-smokers | 92.7% | 90.3% | 87.8% | 86.1% |
| Smokers | 7.3% | 9.7% | 12.2% | 13.9% |
| Physical activity; daily | 18.4% | 17.0% | 15.9% | 16.4% |
| Physical activity; 3-5 times/week | 34.1% | 31.5% | 30.9% | 27.3% |
| Physical activity; 1-2 times/week | 26.7% | 27.2% | 27.9% | 22.7% |
| Physical activity; <1 times/week | 12.8% | 14.8% | 16.5% | 22.1% |
| Physical activity; never | 8.0% | 9.5% | 8.8% | 11.5% |
| Insulin method; injection | 79.7% | 82.7% | 84.5% | 87.2% |
| Insulin method; pump | 20.3% | 17.3% | 15.5% | 12.8% |
| ASA; no | 99.3% | 99.0% | 99.0% | 98.6% |
| ASA; yes | 0.7% | 1.0% | 1.0% | 1.4% |
| Antihypertensive; no | 97.4% | 96.1% | 95.5% | 91.8% |
| Antihypertensive; yes | 2.6% | 3.9% | 4.5% | 8.2% |
| Lipid lowering drug; no | 98.4% | 96.8% | 93.3% | 88.0% |
| Lipid lowering drug; yes | 1.6% | 3.2% | 6.7% | 12.0% |
| Normal blood pressure, Systolic <140mmHg | 97.1% | 95.5% | 95.6% | 94.3% |
| Hypertension,  Systolic >140 mmHg | 2.9% | 4.5% | 4.4% | 5.7% |
| eGFR, ml/min | 111 (97 – 127) | 110 (96 – 127) | 113 (98 – 130) | 118 (102 – 136) |
| eGFR < 30 | 0.07% | 0.06% | 0.11% | 0.00% |
| 30 ≤ eGFR < 45 | 0.05% | 0.03% | 0.11% | 0.27% |
| 45 ≤ eGFR < 60 | 0.30% | 0.23% | 0.11% | 0.00% |
| 60 ≤ eGFR < 90 | 13.94% | 15.57% | 12.67% | 9.84% |
| eGFR ≥ 90 | 85.64% | 84.11% | 87.00% | 89.89% |

ASA, Acetylsalicylic acid; BMI, Body mass index; eGFR, Estimated glomerular filtration rate; HbA1c, Glycated hemoglobin 1c; HDL-Cholesterol, High-density lipoprotein-Cholesterol; LDL-Cholesterol, Low-density lipoprotein-Cholesterol. Yrs, years. ^*^isoBMI was calculated for individuals with age under 18 years according to reference [1]. Continuous variables are presented as medians and interquartile range and categorical variables as proportions.

# **Table S2.** Baseline characteristics of the nephropathy cohort (n=12350) into 4 categories of LDL-cholesterol levels.

|  | LDL <2.6 | 2.6 ≤LDL< 3.4 | 3.4 ≤LDL< 4.1 | LDL ≥ 4.1 |
| --- | --- | --- | --- | --- |
| Number | 7348 | 3477 | 1080 | 445 |
| Age, yrs | 21 (19-24) | 21 (19-27) | 21 (19-27) | 22 (19-28) |
| Females | 40.8% | 45.6% | 45.6% | 45.6% |
| Males | 59.2% | 54.4% | 54.4% | 54.4% |
| Debut age, yrs | 15 (10-20) | 16 (10-22) | 16 (11 – 23) | 16 (10-23) |
| 0-10 yrs | 21.0% | 17.6% | 19.0% | 18.9% |
| 10-15 yrs | 27.8% | 24.3% | 24.6% | 22.7% |
| 15-20 yrs | 24.0% | 22.7% | 18.5% | 19.9% |
| 20-25 yrs | 17.1% | 21.2% | 19.4% | 18.6% |
| 25-30 yrs | 10.1% | 14.2% | 18.5% | 19.9% |
| Duration, yrs | 3.9 (3.6-4.3) | 4.8 (4.5-5.2) | 5.6 (5.3-5.9) | 6.6 (6.2-7.1) |
| Follow-up time, yrs | 82.3% | 19.1% | 0.6% | 0.2% |
| Number of LDL-cholesterol measurements per individual | 5 (3 – 8) | 5 (3 – 8) | 5 (3 – 8) | 5 (3 – 7) |
| HDL-cholesterol, mmol/L | 1.5 (1.2 – 1.8) | 1.4 (1.2 – 1.6) | 1.4 (1.1 – 1.6) | 1.3 (1.1 – 1.5) |
| < 1.1 | 11.1% | 14.9% | 18.5% | 20.7% |
| ≥ 1.1 | 88.9% | 85.1% | 81.5% | 79.3% |
| Total-cholesterol, mmol/L | 3.9 (3.6 – 4.3) | 4.8 (4.5 – 5.2) | 5.6 (5.3 – 5.9) | 6.6 (6.2 – 7.1) |
| < 4.5 | 82.3% | 19.1% | 0.6% | 0.2% |
| ≥ 4.5 | 17.7% | 80.9% | 99.4% | 99.8% |
| Triglyceride, mmol/L | 0.8 (0.6 – 1.1) | 1.0 (0.7 – 1.3) | 1.1 (0.8 – 1.7) | 1.4 (1.0 – 2.2) |
| < 1.7 | 90.5% | 84.6% | 73.8% | 58.4% |
| ≥ 1.7 | 9.5% | 15.4% | 26.2% | 41.6% |
| HbA1c, mmol/mol | 60 (51 – 70) | 62 (53 – 73) | 66 (55 – 76) | 70 (58 – 84) |
| < 48 | 18.2% | 15.2% | 12.1% | 11.0% |
| 48 ≤ to < 58 | 26.1% | 21.5% | 19.7% | 13.7% |
| 58 ≤ to < 70 | 30.8% | 31.3% | 29.2% | 24.5% |
| ≥ 70 | 24.9% | 32.0% | 39.0% | 50.8% |
| ^*^BMI (Kg/m^2^) | 22.8 (20.8 – 25.2) | 23.8 (21.6 – 26.6) | 24.8 (22.2 – 28.2) | 25.5 (22.4 – 29.0) |
| Normal 18.5-24.9 | 72.3% | 61.2% | 51.3% | 44.7% |
| Overweight 25-29.9 | 22.0% | 27.6% | 32.7% | 35.1% |
| Obese ≥ 30 | 5.7% | 11.2% | 16.0% | 20.2% |
| Non-smokers | 92.8% | 90.3% | 88.6% | 86.5% |
| Smokers | 7.2% | 9.7% | 11.4% | 13.5% |
| Physical activity; daily | 17.6% | 17.7% | 14.4% | 16.2% |
| Physical activity; 3-5 times/week | 35.3% | 31.6% | 31.7% | 29.0% |
| Physical activity; 1-2 times/week | 27.2% | 27.8% | 28.7% | 22.0% |
| Physical activity; <1 times/week | 12.2% | 14.2% | 16.0% | 19.1% |
| Physical activity; never | 7.7% | 8.7% | 9.2% | 13.7% |
| Insulin method; injection | 78.0% | 80.4% | 82.8% | 83.4% |
| Insulin method; pump | 22.0% | 19.6% | 17.2% | 16.6% |
| ASA; no | 99.3% | 99.2% | 99.5% | 98.4% |
| ASA; yes | 0.7% | 0.8% | 0.5% | 1.6% |
| Antihypertensive; no | 97.7% | 96.7% | 96.5% | 93.0% |
| Antihypertensive, yes | 2.3% | 3.3% | 3.5% | 7.0% |
| Lipid lowering drug, no | 98.4% | 97.6% | 95.9% | 87.0% |
| Lipid lowering drug, yes | 1.6% | 2.4% | 4.1% | 13.0% |
| Normal blood pressure, Systolic <140mmHg | 96.4% | 95.4% | 95.6% | 92.6% |
| Hypertension,  Systolic >140 mmHg | 3.6% | 4.6% | 4.4% | 7.4% |
| eGFR, ml/min | 111 (97 – 127) | 110 (97 – 128) | 114 (98 – 131) | 118 (101 – 136) |
| eGFR < 30 | 0.04% | 0.03% | 0.00% | 0.00% |
| 30 ≤ eGFR < 45 | 0.05% | 0.03% | 0.09% | 0.22% |
| 45 ≤ eGFR < 60 | 0.26% | 0.23% | 0.09% | 0.00% |
| 60 ≤ eGFR < 90 | 13.91% | 13.86% | 12.23% | 10.57% |
| eGFR ≥ 90 | 85.74% | 85.85% | 87.59% | 89.21% |

ASA, Acetylsalicylic acid; BMI, Body mass index; eGFR, Estimated glomerular filtration rate; HbA1c, Glycated hemoglobin 1c; HDL-Cholesterol, High-density lipoprotein-Cholesterol; LDL-Cholesterol, Low-density lipoprotein-Cholesterol. Yrs, years. ^*^isoBMI was calculated for individuals with age under 18 years according to reference [1]. Continuous variables are presented as medians and interquartile range and categorical variables as proportions.

## Figure S1. Flowchart for the studied group. The overlap between cohorts included 10,262 individuals.

SWEDIABKIDS

2000-2017

n=15111

Cases with at least one

micro-, or macroalbuminuria observation

n=22951

Cases with at least one observation on each covariate

n=11024

Cases with at least one observation on each covariate

n=12350

Cases with at least one

LDL-Cholesterol observation

n=16443

Cases with at least one

LDL-Cholesterol observation

n=14823

Cases with at least one retinopathy examination

n=20716

SWEDIABKIDS + NDR

Type 1 diabetes with < 10 years duration

n=26786

NDR

1998-2017

n=19298

## Figure S2. Scatter plots of blood-lipids on logarithmic scales. Each square contains pairwise measurements made at the same time of two lipids on each patient illustrated


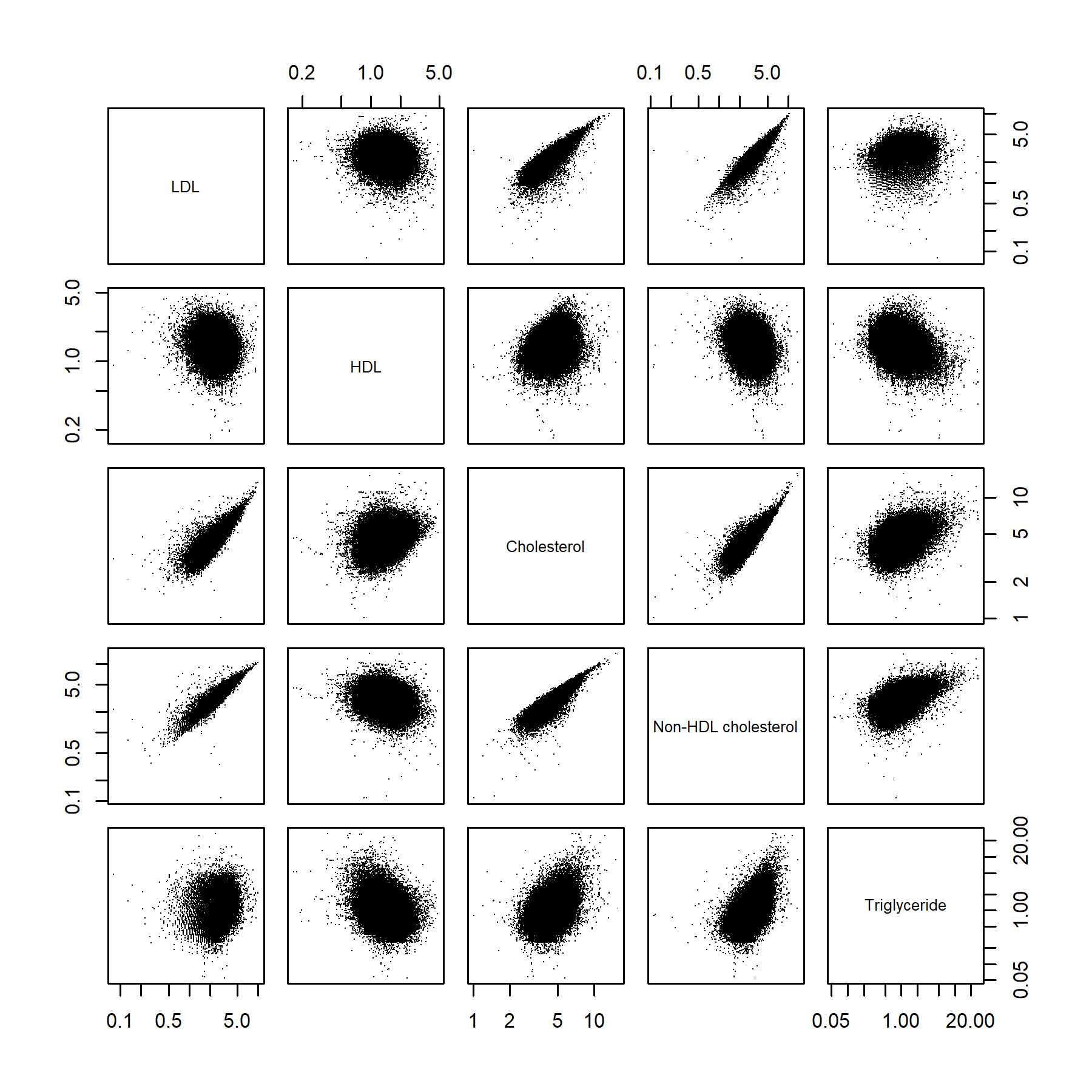


Figure S3. Adjusted hazard ratios for Retinopathy **(A)** and Nephropathy **(B)** in young people with type 1 diabetes, according to baseline LDL-cholesterol level. Beside all covariates that were adjusted for, this forest plot shows no interaction for the known risk factors such as: sex, debut age, HbA1c, BMI, smoking or insulin method used. (Low-density-lipoprotein, LDL) **A)**


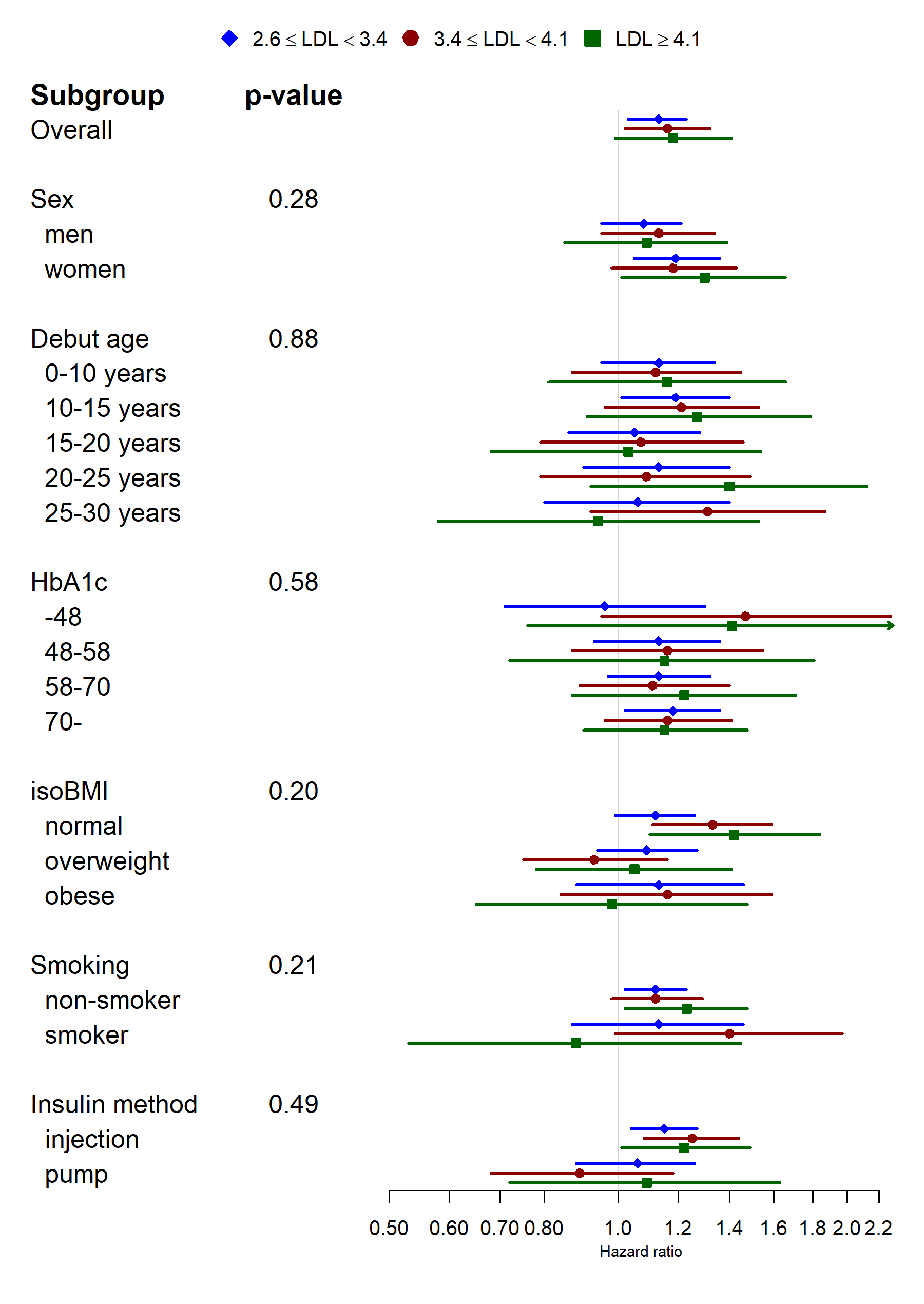


**B)**

**
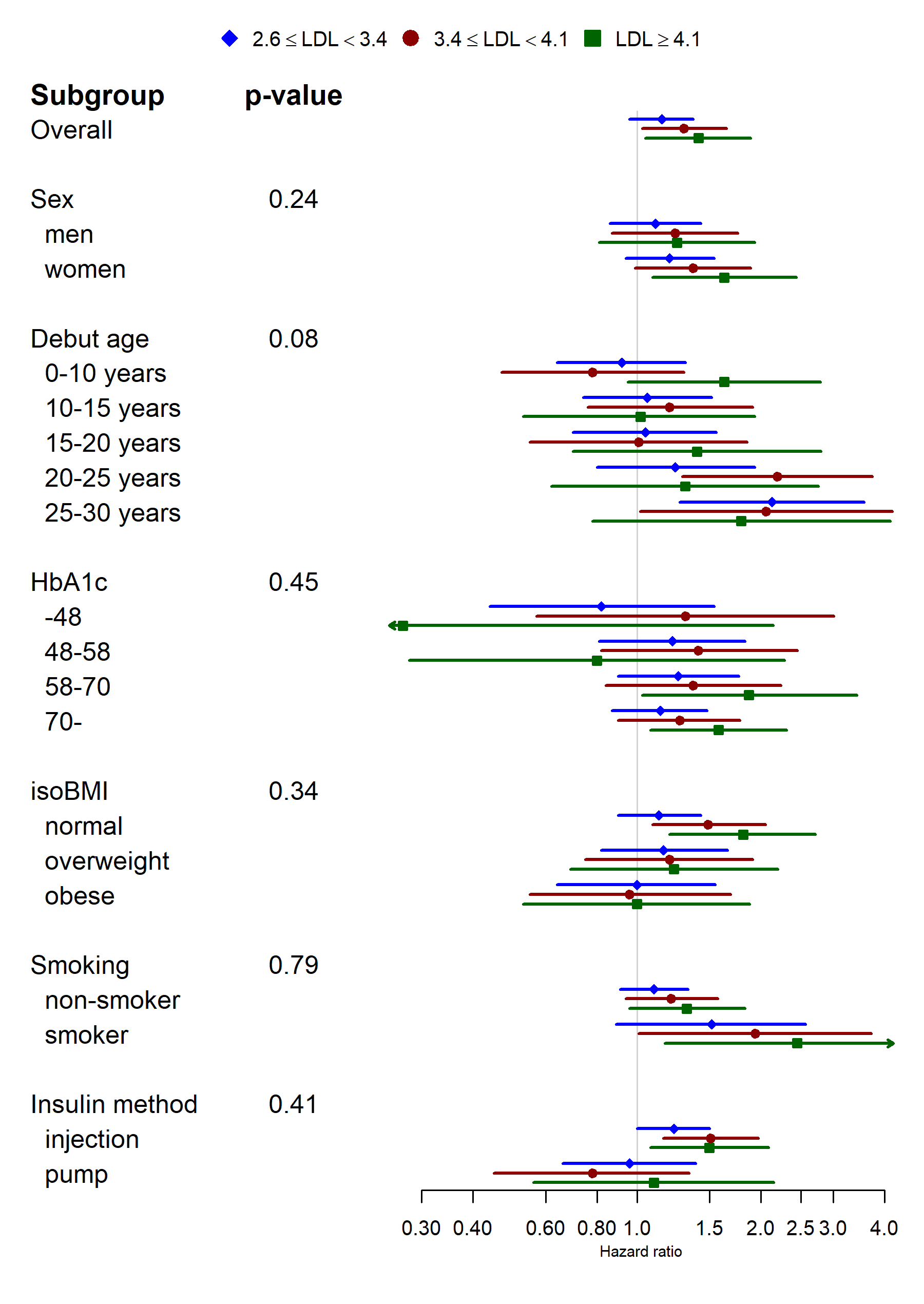
**

**Figure S4**. Relationship between LDL-cholesterol levels, as a continuous variable, and risk of Retinopathy (A) and Nephropathy (B). LDL-cholesterol level was modeled with restricted cubic splines with 4 knots (at 1.25, 3.28, 5.30 and 7.33) in a Cox regression model. The reference level was set at 2.6 mmol/L for the estimation of hazard ratios. For every 0.5 mmol/L increase in LDL-cholesterol level (above reference) HR increased with 1.03 (95% CI: 1.00-1.06), and 1.09 (95% CI: 1.04-1.15) for retinopathy and nephropathy, respectively. (95% confidence bands are presented as blue shaded area).

**A)**


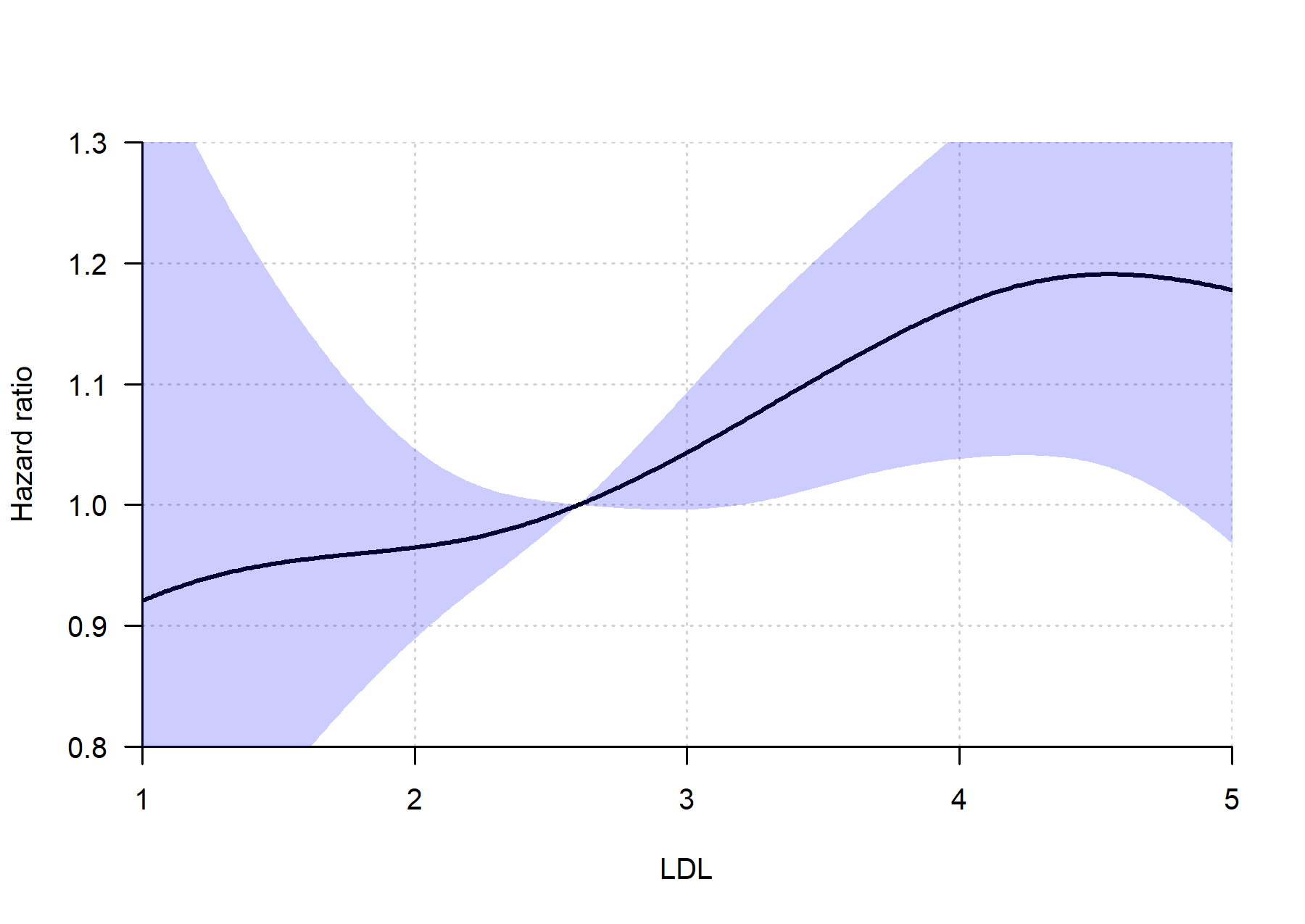


**B)**


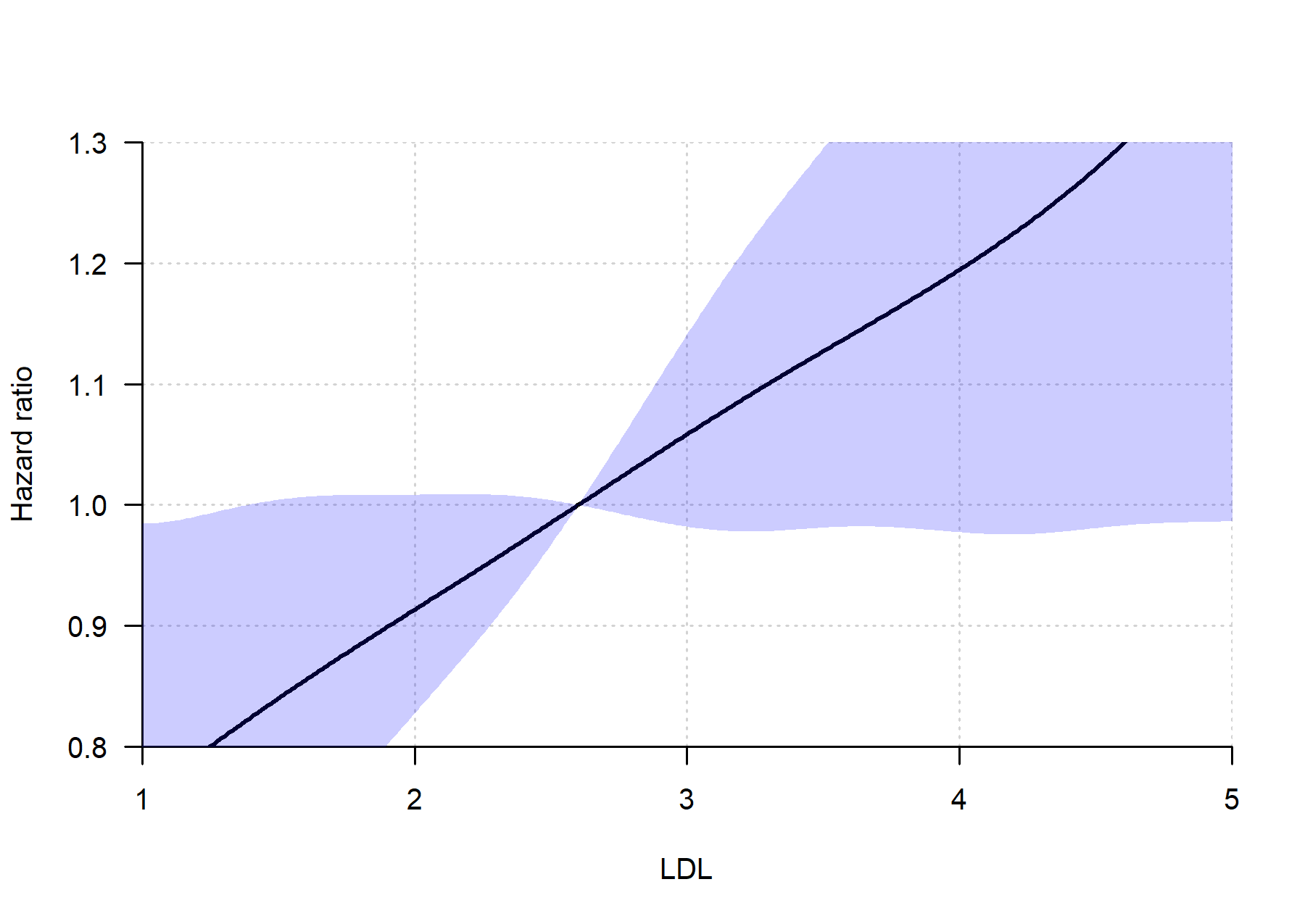


# **Reference**

1. Cole TJ, Bellizzi MC, Flegal KM, Dietz WH. Establishing a standard definition for child overweight and obesity worldwide: international survey. BMJ. 2000;320(7244):1240-3. doi: 10.1136/bmj.320.7244.1240. PubMed PMID: 10797032; PubMed Central PMCID: PMCPMC27365.
